# Validation of an electron Monte Carlo dose calculation algorithm in the presence of heterogeneities using EGSnrc and radiochromic film measurements

**DOI:** 10.1120/jacmp.v12i4.3392

**Published:** 2011-11-15

**Authors:** Jean‐François Aubry, Hugo Bouchard, Igor Bessières, Frédéric Lacroix

**Affiliations:** ^1^ Département de Radio‐Oncologie Centre hospitalier de l'Université de Montréal (CHUM) Montréal; ^2^ Département de Physique Université de Montréal Montréal Québec Canada

**Keywords:** electron dosimetry, macro Monte Carlo, radiochromic film dosimetry, heterogeneous dose calculation

## Abstract

The purpose of this study is to validate Eclipse's electron Monte Carlo algorithm (eMC) in heterogeneous phantoms using radiochromic films and EGSnrc as a reference Monte Carlo algorithm. Four heterogeneous phantoms are used in this study. Radiochromic films are inserted in these phantoms, including in heterogeneous media, and the measured relative dose distributions are compared to eMC calculations. Phantoms A, B, and C contain 1D heterogeneities, built with layers of lung‐ (phantom A) and bone‐ (phantoms B and C) equivalent materials sandwiched in Plastic Water. Phantom D is a thorax‐anthropomorphic phantom with 2D lung heterogeneities. Electron beams of 6, 9, 12 and 18 MeV from a Varian Clinac 2100 are delivered to these phantoms with a $10\times 10\;{\text{cm}}^{2}$ applicator. Monte Carlo simulations with an independent algorithm (EGSnrc) are also used as a reference tool for two purposes: (1) as a second validation of the eMC dose calculations, and (2) to calculate the stopping power ratio between radiochromic films and bone medium, when dose is measured inside the heterogeneity. Percent depth dose (PDD) film measurements and eMC calculations agree within 2% or 3 mm for phantom A, and within 3% or 3 mm for phantoms B and C for almost all beam energies. One exception is observed with phantom B and the 6 MeV, where measured PDDs and those calculated with eMC differ by up to 4 mm. Gamma analysis of the measured and calculated 2D dose distributions in phantom D agree with criteria of 3%, 3 mm for 9, 12, and 18 MeV beams, and criteria of 5%, 3 mm for the 6 MeV beam. Dose calculations in heterogeneous media with eMC agree within 3% or 3 mm with radiochromic film measurements. Six (6) MeV beams are not modeled as accurately as other beam energies. The eMC algorithm is suitable for clinical dose calculations involving lung and bone.

PACS numbers: 87.10.Rt, 87.55.km

## I. INTRODUCTION

Monte Carlo simulation of radiation transport is widely accepted as the most accurate method to calculate dose distributions of photon and electron beams.^(^ ^1^ ^,^ ^2^ ^)^ MC algorithms simulate the basic physical laws governing the interactions of particles with matter in a stochastic manner. However, the routine use of MC algorithms for external beam radiotherapy has not yet entered the world of radiation oncology clinics, mainly because of the long calculation times required by these simulations to obtain statistically accurate dose distributions. Recently a partial Monte Carlo solution has been released commercially for electron beam dose calculations, where approximations in the radiation transport simulation accelerate calculation time.^(^ ^3^ ^)^ This method is referred to as the macro Monte Carlo algorithm (MMC).^(^ ^3^ ^)^ MMC uses Monte Carlo precalculated probability distributions to limit the computational cost associated with particle transport. MMC is marketed under the trade name electron Monte Carlo (eMC), and is available on the Eclipse treatment planning system (Varian Medical Systems, Palo Alto, CA). Another vendor has also released a voxel‐based Monte Carlo calculation platform (Oncentra MasterPlan, Nucletron Inc., Veenendaal, The Netherlands) for electron beams derived from VMC++.^(^ ^4^ ^,^ ^5^ ^)^ The goal of this study is to evaluate the accuracy of the eMC algorithm in heterogenous media.

A small number of studies have already been published on the validity of this commercially available algorithm. The emphasis is generally on the verification of output factors and dose distributions in water.^(^ ^6^ ^,^ ^7^ ^)^ However, Monte Carlo simulations are especially advantageous in conditions of medium heterogeneity and irregular surfaces where full electron transport simulation is necessary to accurately calculate dose distributions. Pencil beam‐based algorithms can show large differences to measured doses in these conditions.^(^ ^8^ ^,^ ^9^ ^)^ This is due to assumptions inherent to the algorithm, such as the modeling of electron scatter with a Gaussian distribution and the lack of delta ray and Bremsstrahlung modeling. Some authors have already reported on the accuracy of macro Monte Carlo algorithms in the presence of heterogeneous media;^(^ ^10^ ^–^ ^13^ ^)^ however, measurements were limited to regions located before or after the heterogeneity. Neuenschwander et al.^(^ ^3^ ^)^ performed validation simulations of MMC within bone and lung heterogeneity by comparing MMC results to EGS4 results. The agreement between MMC and EGS4 was generally within 2.5% at 20 MeV and 3.5% at 10 MeV, and of 5% at the bone–water interface at 10 MeV for a heterogeneous water and bone phantom. To the best of our knowledge, there are no validation studies incorporating dose measurements in a clinical electron beam (i.e., inside the heterogeneities themselves – although they were done in presence of heterogeneities), nor of situations combining irregular surfaces and heterogeneities. Such irradiation conditions, nonetheless, can occur everyday in a radiation oncology clinic.

In order to obtain accurate measurements in these conditions, a dose detector has to be small enough to be inserted into the inhomogeneous regions, has to have an excellent spatial resolution and has to have no energy dependence and, furthermore, the detector must not perturb the electron fluence. One detector that fulfills these requirements is radiochromic film.^(^ ^14^ ^–^ ^16^ ^)^ With the use of radiochromic film, the accuracy of the eMC algorithm can be experimentally validated. This paper presents the results of a validation study of eMC dose calculations involving heterogeneities (bone and lung) using radiochromic film dosimetry (GAFCHROMIC EBT, International Specialty Products, Inc., Wayne, NJ).

## II. MATERIALS AND METHODS

### A. The electron Monte Carlo algorithm

The eMC algorithm as implemented in the Eclipse treatment planning software (TPS) has two major components:^(^ ^5^ ^)^ 1) an electron beam phase space of particles located at the end of the applicator,^(^ ^17^ ^)^ and 2) a transport and dose deposition algorithm based on precalculated Monte Carlo simulations.^(^ ^5^ ^)^

The initial phase space is based on a model of the linear accelerator and then adjusted to match actual machine measurements.^(^ ^17^ ^)^ The required input data for the TPS to create an electron beam are obtained from open field measurements (taken without an applicator and with jaws wide open) and applicator field measurements. For each beam of a given energy, open field measurements of the following must be provided:

- percent depth dose (PDD) in water at a source to surface distance (SSD) of 100 cm
- absolute dose per monitor unit (MU) at a point along the PDD curve
- profile in air at a distance of 95 cm from the linac source

Also, for each energy and applicator combination, the following measurements are required:

- PDD in water at 100 cm SSD
- absolute dose per monitor unit (MU) at a point along the PDD curve

The electron transport and dose deposition processes are computed using probability tables of presimulated results, which are obtained with mono‐energetic electrons entering a uniform sphere of a given material.^(^ ^5^ ^)^ By precalculating such tables for different electron energies, sphere radii and material, electron transport and dose deposition in heterogeneous media can be simulated in fewer, faster steps than a full EGS4/EGSnrc Monte Carlo simulation — hence the name macro Monte Carlo. In doing so, however, approximations are used concerning the transport of secondary electrons.

When an electron gets close to an interface between different media, precalculated results from spheres smaller than the distance to the interface (down to 0.5 mm) will be used so that interface effects can be modeled more accurately. The algorithm can also stop a particle at the interface and restart the transport simulation with a more appropriate probability table. The eMC algorithm has presimulated results from five different sphere materials, namely air $(0.001205\;g/{\text{cm}}^{3})$, lung phantom LN4 $\left(0.3\;g/{\text{cm}}^{3}\right)$, water $\left(1\;g/{\text{cm}}^{3}\right)$, Lucite $\left(1.19\;g/{\text{cm}}^{3}\right)$, and solid bone phantom SB3 $(1.84\;g/{\text{cm}}^{3})$. When tissues of intermediate density are found, the material closest to the actual CT‐measured density is used, and the energy deposited is scaled according to the ratio of restricted stopping powers between the current medium and the one used in the precalculated tables. The eMC algorithm, therefore, always calculates dose to the medium, as opposed to dose in water‐equivalent materials of varying density.

For a given calculation, the options that are available to the user are: dose voxel size, percentage level of desired uncertainty, type of dose distribution smoothing (none, 2D or 3D Gaussian), level of smoothing (low, medium, or high), and seed number used for the random number generator.

The uncertainty level is defined as the average statistical uncertainty of all voxels receiving at least 50% of the maximum dose. Thus, if a 1% uncertainty is requested, in the end any given voxel can have more than 1% uncertainty as long as the general criterion is met. The statistical uncertainty of all voxels receiving less than 50% of the maximum dose remains hidden from the user.

Version 8.1.17 of the eMC algorithm is used for all calculations throughout this study.

### B. Radiochromic film dosimetry

GAFCHROMIC EBT films are used for relative dosimetry. The films are scanned prior and after irradiation using an EPSON 10000XL flatbed scanner in opaque mode at 150 dpi and 48 bit RBG format. All films are scanned five times both before and after irradiation, and the scans are averaged to minimize noise. A scanner heterogeneity correction is then applied to the averaged images, similar to the one described by Saur and Frengen.^(^ ^18^ ^)^ After irradiation all films are scanned after at least 16 hours so that the optical density is stable. Care is taken that the temperature and humidity are kept constant while scanning.

An optical density to dose calibration curve is fitted using 13 measurement pairs of absolute dose (measured with an ion chamber) and optical density. The fitting procedure and dose uncertainty analysis follow the method described in Bouchard et al.^(^ ^19^ ^)^ and are summarized here for the sake of completeness. Film calibration is carried out by exposing 12 pieces of radiochromic film to doses from 0 to 800 cGy. Each piece of film is calibrated separately in a solid water phantom. A calibrated ion chamber is located in the solid water stack under the film. A least squares fit is performed with these measurements and a mathematical functional form that minimizes the statistical uncertainty of the procedure. Moreover, the correlation between fit parameters is taken into account to produce a more accurate estimate of the statistical uncertainty of the measurements. Image processing and calibration is performed using an in‐house software developed with MATLAB (The MathWorks, Inc., Boston, MA). Using this software, point dose values are either extracted directly from the scanned images or planar dose images are exported to the RIT113 software (Radiological Imaging Technology, Inc., Colorado Springs, CO) for further analysis.

### C. Phantoms with 1D heterogeneities

#### C.1 Phantom descriptions

Three phantoms with heterogeneity in the depth direction are designed for validation purposes. These phantoms are built from slabs of heterogeneous material sandwiched in water‐equivalent slabs. The first (phantom A) is a “lung” phantom made of 1.5 cm of Plastic Water slabs (CIRS, Norfolk, VA), followed by a combination of lung‐equivalent slabs adding up to 9 cm, and ending with 5 cm of Plastic Water. The lung equivalent slabs are also manufactured by CIRS and have a $0.21\;g/{\text{cm}}^{3}$ physical density.

Two “bone” phantoms are also used for this study. One (phantom B) is composed of 1 cm of Plastic Water slabs, then bone‐equivalent slabs totaling 1 cm, and finally 5 cm of Plastic Water. The bone equivalent slabs were SB3 cortical bone model #450 from Gammex Inc. (Middleton, WI), with a physical density of $1.82\;g/{\text{cm}}^{3}$. Phantom C is similar to phantom B, except that 2 cm of SB3 bone equivalent slabs were used instead of 1 cm.

#### C.2 Phantom irradiation

A Varian Clinac 2100 linear accelerator (Varian Medical Systems, Palo Alto, CA) is used to deliver radiation to radiochromic films inserted in phantoms A, B and C. Electron beams of 6, 9, 12, and 18 MeV are commissioned on this machine and calibrated to give 1 cGy per monitor unit (MU) at the depth of maximum dose $\left(d_{\max}\right)$.

Lung phantom A is irradiated with electron beams of 6, 9, 12, and 18 MeV, at 100 cm SSD with a $10\times 10\;{\text{cm}}^{2}$ field and a gantry angle of 0°; 250 MU are given for each beam energy.

Bone phantoms B and C are irradiated with different beam energies. Phantom B is used for electron beam energies of 6 and 9 MeV because of the shorter range of these beams, while phantom C is irradiated with beams of 12 and 18 MeV. For all four beams, the phantoms are setup at 100 cm SSD and irradiated with a $10\times 10\;{\text{cm}}^{2}$ field using 250 MU.

#### C.3 Radiochromic film measurements

In the lung phantom (A), squares of radiochromic film of $2\times 2\;{\text{cm}}^{2}$ are inserted between certain slabs, centered along the beam central axis, at depths of 1.0, 1.5, 2.5, 3.5, 6.5, and 9.5 cm. For a given beam energy, all the films are irradiated at the same time.

In bone phantoms B and C, radiochromic films of $2\times 2\;{\text{cm}}^{2}$ are placed along the beam axis at depths of 0.5, 1.0, 1.3, and 1.5 cm for all beam energies. Moreover, extra films are inserted at 2.5 cm depth for the 6 MeV beam; 3.0 cm for the 9 MeV beam; 2.5, 2.8, and 4.0 cm for the 12 MeV beam; and 2.5, 2.8, and 5.0 cm for the 18 MeV beam. Similarly to phantom A, all films used for one beam energy are irradiated simultaneously.

Absolute dose measurements are obtained from the radiochromic films using the MATLAB user code described in Section B above. The dose reading from the square films is taken from a small area of about $0.5\;\times \;0.5\;{\text{cm}}^{2}$ in the center of the films. The size of the region of interest (ROI) is chosen according to the dose calculation resolution, and uncertainties with regards to the size of the ROI are carefully considered in the estimations.^(^ ^19^ ^)^ The measurements are normalized so the dose is 100% at 1.0 cm depth for phantom A and at 0.5 cm depth for phantoms B and C.

#### C.4 eMC dose calculations

The three phantoms are created in Eclipse using contouring tools and setting the HU of each layer contour so that its density corresponds to the manufacturer's specifications. The irradiation conditions described above are reproduced on the TPS, and the resulting dose calculation grids are exported to MATLAB in DICOM format. PDDs are then extracted from the dose grids and normalized to 100% at 1.0 cm depth for phantom A and at 0.5 cm depth for phantoms B and C.

For all these calculations, the voxel size is set to 1 mm, the uncertainty level to 1%, and a medium 3D Gaussian smoothing is requested to reduce stochastic noise.

#### C.5 Monte Carlo simulations

Monte Carlo simulations are used for two purposes. Firstly, radiochromic film measurements can be reported accurately as dose to medium only when scaling with the stopping power ratio (SPR) of film to medium. This ratio varies according to the electron energy spectrum, which itself is a function of depth. Calculations of stopping power ratios are performed using Monte Carlo simulations. In this study, the EGSnrc‐based SPRRZnrc user code^(^ ^20^ ^)^ is employed to compute the SPR for phantoms B and C. No SPR corrections are applied to the measurements in lung material since the stopping powers are equivalent to those in water within 1%.^(^ ^21^ ^)^

Monte Carlo simulations are also used in this part of the study as a second validation tool for eMC dose calculations. In these cases, the EGSnrc user code DOSXYZnrc^(^ ^22^ ^)^ is employed to obtain dose‐to‐medium PDDs in phantoms A, B, and C. These PDDs are normalized in an identical fashion to the PDDs calculated with eMC.

For all simulations with SPRZnrc and DOSXYZnrc a precalculated phase space located at the end of the electron beam applicator is used as the particles source. Phase spaces of the four commissioned beam energies on the linac are calculated for the $10\times 10\;{\text{cm}}^{2}$ applicator using the BEAMnrc user code.^(^ ^23^ ^,^ ^24^ ^)^

Radiation transport in the accelerator is modeled using the Monte‐Carlo code BEAMnrc code. The accelerator geometry and materials are obtained from the manufacturer's data for the Varian Clinac 2100 series accelerator. The electron beam is modeled for four energies (6, 9, 12, and 18 MeV) using a $10\;\times \;10\;{\text{cm}}^{2}$ applicator. The accelerator model comprises: the electron exit window, the primary collimator, the primary and secondary scattering foils, the monitor chamber, the mirror, two movable collimator jaws, and the applicator. An ECUT of 0.7 MeV and a PCUT of 0.01 MeV are used. A phase space is scored at 95 cm from the source.

DOSXYZnrc is used to calculate the dose deposited in a water phantom. The model is validated by comparing measured depth doses and in‐plane and cross‐plane profiles at 100 cm SSD for a $10\;\times \;10\;{\text{cm}}^{2}$ field measured with an electron diode. The energy of the electron beam at the electron exit window is not known with sufficient accuracy; therefore, the energy of the beam is modified to match the $R_{50}$ depth of the measured depth doses at 6, 9, 12, and 15 MeV. Measurements are performed with an electron diode, model ${\text{EFD}}^{3G}$ (Scanditronix, Uppsala, Sweden). The resulting incident electron beam energies are 7.1, 10, 13.5, and 16.9 MeV. Three criteria are retained for the comparison of the simulated and measured beam profiles: the relative dose difference in‐field, the mean distance between measured and simulated dose in the penumbra, and the overall shape of the horns. The acceptable dose difference is set to 1%−1 mm. Differences are less than 1% in‐field and less than 1 mm in the penumbra.

### D. Anthropomorphic phantom with 2D heterogeneities

#### D.1 Phantom description

**Figure 1:**
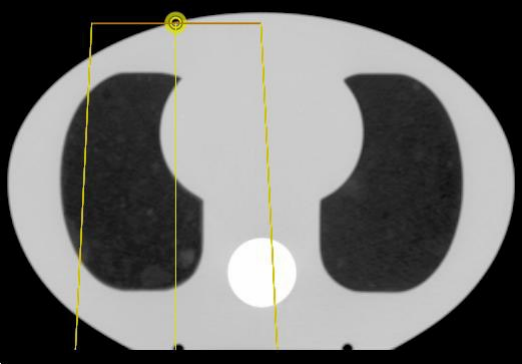
Axial slice of the anthropomorphic phantom used to evaluate 2D heterogeneities. The lateral offset of the electron beam is also shown, the yellow dot representing the beam isocenter.

An anthropomorphic phantom of the thorax section of the human body (Model 002LFC IMRT Thorax Phantom, CIRS, Norfolk, VA) is used to validate dose calculations in a clinically realistic situation. Its maximum height and width are 20 cm and 30 cm respectively, and the section used is 18 cm long. This phantom is primarily made of Plastic Water Diagnostic Therapy (PWDT) material, but also includes two lungs and a bony spine structure (Fig. 1). The lungs are made of the same material used in the design of the 1D phantoms. The bone region is not included in the irradiated volume and, therefore, its composition has no consequence on the dose distribution.

#### D.2 Phantom irradiation

Electron beams of 6, 9, 12, and 18 MeV are delivered to the phantom with a Varian Clinac 2100X linear accelerator. The beam is collimated with a $10\times 10\;{\text{cm}}^{2}$ electron applicator at gantry angle of 0° with the phantom offset laterally by 5 cm from the beam central axis, as shown in Fig. 1. The SSD is set to 100 cm, and 250 MU are delivered by each beam.

#### D.3 Radiochromic film measurements

Radiochromic films are cut so that their shape matches the external contour of the phantom in the axial plane. These films are then inserted in the phantom so that they lie in the same axial plane as the beam central axis. Small metal pieces embedded in the phantom mark three small holes in the films for further registration. Relative planar dose measurements are obtained from the radiochromic films using the procedure and the MATLAB user code described earlier. These dose planes are exported to the RIT software for comparison with calculated dose planes. Analysis is performed using the same technique, as described in Section C.3 above.

#### D.4 eMC dose calculations

A tomographic image of the thorax phantom is obtained with a CT scanner and sent to the TPS. With the help of automatic contouring tools, the HU values of the three materials composing the phantom are overridden so that their densities correspond to their actual values. By forcing the HU and therefore the density of the materials, any problems associated with the stochiometric calibration of our scanner are avoided. The irradiation conditions described in Section D.2 above are then reproduced in the TPS. All the dose calculations are performed with a voxel size of 1 mm and an uncertainty level of 1%, and are smoothed by a 3D Gaussian filter of medium strength. The planar doses located at the beam central axis are exported to the RIT software for comparison with the radiochromic film doses.

## III. RESULTS & DISCUSSION

### A. Phantoms with 1D heterogeneities

#### A.1 Experiments with lung phantom A

**Figure 2:**
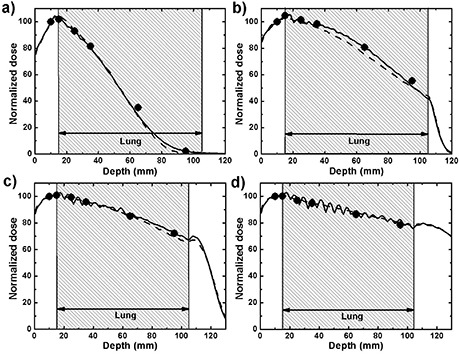
PDDs in phantom A calculated with eMC (solid line) and simulated with EGSnrc (dashed line) compared with radiochromic film measurement (dots): (a) 6 MeV beam, (b) 9 MeV beam, (c) 12 MeV beam, (d) 18 MeV beam. For EGSnrc calculations, uncertainties are less than 0.7%. For radiochromic film, 1σ error bars lie between 0.7% and 1%, which is smaller than the size of the dot.

Figure 2 shows PDDs measured with film, calculated by eMC and simulated with EGSnrc in phantom A for four electron beams. The overall agreement between calculated and measured dose is within 2% or 1 mm for the 6, 9, and 12 MeV beam energies, and within 1% or 2.5 mm for the 18 MeV beam. One exception is the 6 MeV beam, where the greatest difference between the film measurements, eMC, and EGSnrc results is 5.5% or 3 mm, located in the sharp gradient region at a depth of 65 mm. For the 12 MeV and 18 MeV beams, the maximum difference between radiochromic, eMC, and EGSnrc doses is located at 95 mm depth, the deepest point of radiochromic film measurement.

In all calculations, the thickness of the film pieces inserted in the phantom were neglected. This should only affect the results in negligible ways as the thickness of each film layer is only 0.23 mm. Moreover, film pieces of only $2\times 2\;{\text{cm}}^{2}$ are inserted in a $10\times 10\;{\text{cm}}^{2}$ field, and because electrons do not travel in a straight line due to multiple scattering events, the majority of the electron fluence does not travel through the supplemental film thickness.

The percent dose (y‐axis) and depth (x‐axis) uncertainties are not visible on Fig. 2 for the radiochromic film measurements because they are smaller than the dots. The percent dose uncertainty includes the sources of uncertainty listed in Table 1 in a paper by Bouchard et al.^(^ ^19^ ^)^ These uncertainties are evaluated for each measurement by the in‐house software used for radiochromic film dosimetry, and typically range between 0.7% and 1.0%, depending on the exposure dose. In the x‐axis, it is assumed that every measurement is within a ±1 mm uniform distribution, which is equivalent to a standard deviation of 0.6 mm in a normal distribution. In regions of high‐dose gradient, this ±1 mm uniform uncertainty distribution is summed in quadrature with the previously discussed uncertainty in the y‐axis based on the dose gradient. The dose gradient is taken from the eMC calculations. For EGSnrc simulations, over 100 million particles were launched, which results in statistical uncertainties always smaller than 0.7%. eMC calculations are requested at a 1% statistical uncertainty. However, this only applies to dose voxels receiving at least 50% of the maximum dose, and the uncertainty for the rest of the volume is unknown, as explained in Section II A above.

A seemingly periodic oscillation can be clearly observed in the PDDs calculated by eMC for the 12 MeV and 18 MeV beams in the lung region of the phantom. This phenomenon is also present to a lesser degree for the 9 MeV beam. The explanation for this is not straightforward without intimate knowledge of the algorithm's inner workings. In any case, these results are obtained with a medium 3D Gaussian filtering, and although not shown in Fig. 2, any reduction in the strength of the filtering only amplifies this problem, making it difficult to visualize isodoses. The periodic oscillation also seems present in the results of Popple et al. in their Figs. 7 and 8.^(^ ^12^ ^)^ Ding et al.^(^ ^11^ ^)^ have also shown that the use of small voxel size (around 2 mm or less) and Gaussian smoothing produce reasonably accurate and much less noisy results, without observing this oscillation effect in homogeneous media.

**Figure 3:**
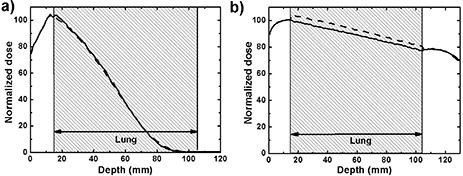
PDDs in phantom A simulated using EGSnrc with LN4 (solid line) and ICRP (dashed line) lung compositions: (a) 6 MeV beam, (b) 18 MeV beam. Uncertainties are less than 0.7%.

For dose calculations in lung medium, the eMC algorithm uses probability distributions functions that were computed in LN4 material, as mentioned in Section II A above. However LN4 is not absolutely equivalent to human lung; the stopping power ratios between LN4 and human lung (as defined by the ICRP Reference Man^(^ ^25^ ^)^) are 1.00 and 0.95 at 10 MeV and 100 MeV, respectively, and the scattering power ratios vary between 0.89 and 0.90.^(^ ^26^ ^)^ Figure 3 shows EGSnrc simulations with 6 MeV and 18 MeV beams using LN4 and ICRP lung compositions, both with identical densities. No difference is observed at 6 MeV, but at 18 MeV the dose in the lung is about 4.5% higher with the ICRP composition, which suggests the mass stopping powers of these materials differ at this particular energy. This is a drawback of calculating dose to the medium, where systematic errors can occur if the composition of a material differs from the materials that have been used to simulate radiation transport.

#### A.2 Experiments with bone phantoms B and C

**Figure 4:**
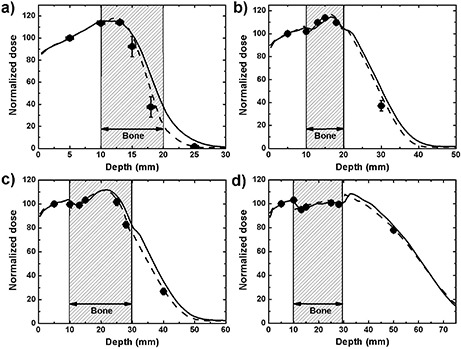
PDDs in phantoms B and C calculated with eMC (solid line) and simulated with EGSnrc (dashed line) compared with radiochromic film measurement (dots): (a) 6 MeV beam in phantom B, (b) 9 MeV beam in phantom B, (c) 12 MeV beam in phantom C, (d) 18 MeV beam in phantom C. For EGSnrc calculations, uncertainties are less than 0.7%. For radiochromic film, 1 σ error bars either lie between 0.7% and 1%, which is smaller than the size of the dot, or are shown explicitly.

The measured, calculated, and simulated PDDs through bone phantoms B and C are shown in Fig. 4 for four beam energies. In the bone region, the radiochromic film dose measurements are scaled by the ratio of stopping powers between bone and water calculated with SPRRZnrc. This correction varies between 11% and 13% through all beam energies, with the higher correction taking place at 6 MeV and the lowest at 18 MeV. The difference in mass stopping powers can be clearly seen at the interface between water and bone with the 12 and 18 MeV beams, where the dose drops abruptly due to the lower mass stopping power of bone.

The results of these experiments are very good, with eMC calculations being within 3% or 3 mm of both film measurements and Monte Carlo simulations, except in the dose falloff and the tail of the 6 MeV PDD. In general the greatest discrepancies between film dose measurements and eMC calculations occur in the gradient regions at lower energies. At 6 MeV, this difference is 30% or 4 mm at a depth of 18 mm, and 19% or 3 mm at 9 MeV and 30 mm depth. At energies of 12 MeV and 18 MeV, the maximum differences are 12% and 5%, respectively, and both under 3 mm. Maximum differences between film doses and EGSnrc results are smaller, being 14% at 6 MeV, 8% at 9 MeV, and 2% at 12 MeV and 18 MeV, while the distance to agreement is always under 3 mm. However, because all these errors occur in very steep dose gradients, distances to agreement are more relevant than dose differences. Also, the difference between full Monte Carlo and macro Monte Carlo simulations can be appreciated in Fig. 4, where the full EGSnrc simulations all match more closely the film measurements than the eMC calculations. The approximations in the eMC algorithm, mainly the presimulation of electron transport and dose deposition and the assumption of unidirectional secondary particles, are shown to have an impact for geometries of the type found in Fig. 4. Smoothing of the dose distribution could possibly play a role in cases like these, since the dose gradients are steeper than in water. Reducing the smoothing level would, however, bring other disadvantages, such as a noisier dose distribution.^(^ ^11^ ^)^

The uncertainties for these experiments are all similar to the ones obtained with the lung phantom and noted in the Results & Discussion Section A.1 above, with the only difference being that the dose gradients can be much steeper in bone than lung, causing bigger positioning uncertainties in the y‐axis in these regions, as shown in Fig. 4.

The 6 MeV beam shows the largest discrepancies between measured and calculated doses, which is expected because of steeper dose gradients and the limited ability of the algorithm to accurately model this beam even in homogeneous conditions. Of all the electron beam energies modeled in our TPS, the 6 MeV beam matches with more difficulty the measured model input data. For example, our own validation work shows that the calculated PDD in water for this beam energy with a $10\times 10\;{\text{cm}}^{2}$ applicator at 100 cm SSD has a depth of 50% of maximum dose $\left(R_{50}\right)$, 1.8 mm deeper than the value measured with an electron diode. It is therefore expected that calculations in more complex situations would not show a better agreement than that. The release notes for newer versions of the algorithm (8.6 and above) specify that improvements in the beam model were made for beams of low energy; however, such verification lies outside of the scope of this paper.

Another point of interest regarding these results is the effect of the dose voxel size. The eMC PDDs shown in Fig. 4 were calculated using a 1 mm voxel size, which is the finest resolution available in eMC. The agreement between film measurement, Monte Carlo simulations, and eMC can deteriorate with greater voxel sizes. For example, the $R_{50}$ of the 6 MeV beam calculated by eMC and shown in Fig. 4 shifts 1.8 mm deeper using 2.5 mm voxel size instead of 1 mm. Varying the strength of the smoothing filter did not affect the calculations in this case. While a complete study of the effect of grid size is beyond the scope of this paper, caution should be exerted in the selection of the dose voxel size when calculating dose distributions with very sharp dose gradients, which can occur in bone medium, for example. A study by Ding et al.^(^ ^11^ ^)^ has already shown that steep lateral dose gradients caused by heterogeneities are correctly modeled using 2 mm voxels, but not with 5 mm voxels.

### B. Anthropomorphic phantom with 2d heterogeneities

**Figure 5:**
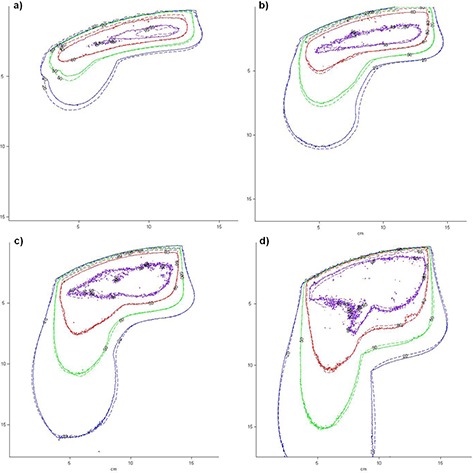
Isodose levels of 95%, 80%, 50%, and 20% obtained in the anthropomorphic phantom: (a) 6 MeV beam, (b) 9 MeV beam, (c) 12 MeV beam, (d) 18 MeV beam. The solid line represents radiochromic film measurements and the dashed line eMC calculations.

Relative isodoses measured with radiochromic films and calculated with eMC in the anthropomorphic thorax phantom are compared in Fig. 5 for beam energies of 6, 9, 12, and 18 MeV. Isodose lines of 95%, 80%, 50%, and 20% are shown. The normalization between the measured and calculated dose planes is tweaked to maximize the overall agreement of these four isodose lines. For energies of 9, 12, and 18 MeV, the isodose lines match up extremely well, except in the buildup region and the interface between lung and plastic water than runs parallel to the beam axis. This is expected, as even Monte Carlo simulations struggle to accurately model electron transport when the incident beam is parallel to an interface. For the 6 MeV beam, discrepancies can be seen in the buildup region, in some of the high‐dose region, and in the lower dose region in the lung section of the phantom. In this case the maximum distance between measured and calculated isodose lines is 4 mm, between the 20% isodoses in the lung.

**Figure 6:**
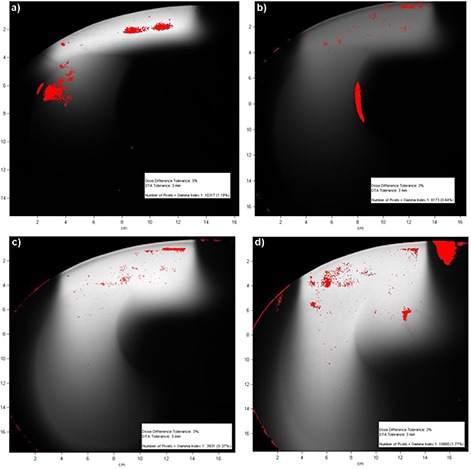
Gamma analysis between radiochromic film measurements and eMC calculations in the anthropomorphic phantom, with 3%, 3 mm criteria: (a) 6 MeV beam, (b) 9 MeV beam, (c) 12 MeV beam, (d) 18 MeV beam. Areas where the criteria were not met are shown in color.

Figure 6 shows the areas where a gamma analysis with criteria of 3% and 3 mm failed for the four same dose distributions. For the 6 MeV beam, there are two such areas, one around the region of maximum dose in water, and one in the gradient region in the lung. This suggests that the eMC algorithm does not handle the irregular surface of the phantom as well for the 6 MeV beam as it does for other energies. A gamma analysis with criteria of 5% and 3 mm yields no dose points above these limits (not shown). The 9 MeV beam gamma analysis shows a small region of gamma values failing the test near the deep lung–water interface, as discussed previously. For the 12 MeV and 18 MeV dose distributions, the pixels failing the gamma analysis can either be attributed to noise in the calculation, or they lie outside the field.

## IV. CONCLUSIONS

Radiochromic film dosimetry and Monte Carlo simulations are used to validate dose distributions calculated with the commercially available eMC algorithm. Calculations and measurements in phantoms with lung and bone heterogeneities along one and two dimensions are performed. For beam energies of 9, 12, and 18 MeV, the dose calculated by eMC in heterogeneous media and beyond is within 3% or 3 mm and often better. For the 6 MeV beam, the measured and calculated doses are within 5% or 4 mm. The eMC algorithm is suitable for clinical dose calculations involving heterogeneous media such as lung and bone.

## ACKNOWLEDGMENTS

The authors would like to thank Robert Doucet for revising the manuscript, and Joannie Desroches for her help with radiochromic film measurements.
